# Remarkable Rates of Lightning Strike Mortality in Malawi

**DOI:** 10.1371/journal.pone.0029281

**Published:** 2012-01-09

**Authors:** Monique Borgerhoff Mulder, Lameck Msalu, Tim Caro, Jonathan Salerno

**Affiliations:** 1 Department of Anthropology, University of California, Davis, California, United States of America; 2 Mpamba Health Center PA Mpamba, Nkhata Bay, Malawi; 3 Department of Wildlife, Fish and Conservation Biology and Center for Population Biology, University of California, Davis, California, United States of America; 4 Graduate Group in Ecology, University of California, Davis, California, United States of America; 5 Wissenschaftskolleg zu Berlin, Germany; Indiana University, United States of America

## Abstract

Livingstone's second mission site on the shore of Lake Malawi suffers very high rates of consequential lightning strikes. Comprehensive interviewing of victims and their relatives in seven Traditional Authorities in Nkhata Bay District, Malawi revealed that the annual rate of consequential strikes was 419/million, more than six times higher than that in other developing countries; the rate of deaths from lightning was 84/million/year, 5.4 times greater than the highest ever recorded. These remarkable figures reveal that lightning constitutes a significant stochastic source of mortality with potential life history consequences, but it should not deflect attention away from the more prominent causes of mortality in this rural area.

## Introduction

Consequential lighting strikes (CLS), defined as lightning events causing physical injury, unconsciousness or death to humans, result in 0.3 to 6 fatalities/million people/annum in developed and developing countries, respectively [1]. Visiting Old Bandawe, David Livingstone's second mission settlement on the northwest shore of Lake Malawi [2], we noted many funerals resulting from fatal lightning strikes. Given that 78% of all lightning events occur in the tropics [3], the possibility of high localized risk of CLS in parts of Central Africa merits investigation because it has ramifications for life history theory, public safety concerns, and development strategies on the African continent.

## Results

**Figure 1:**
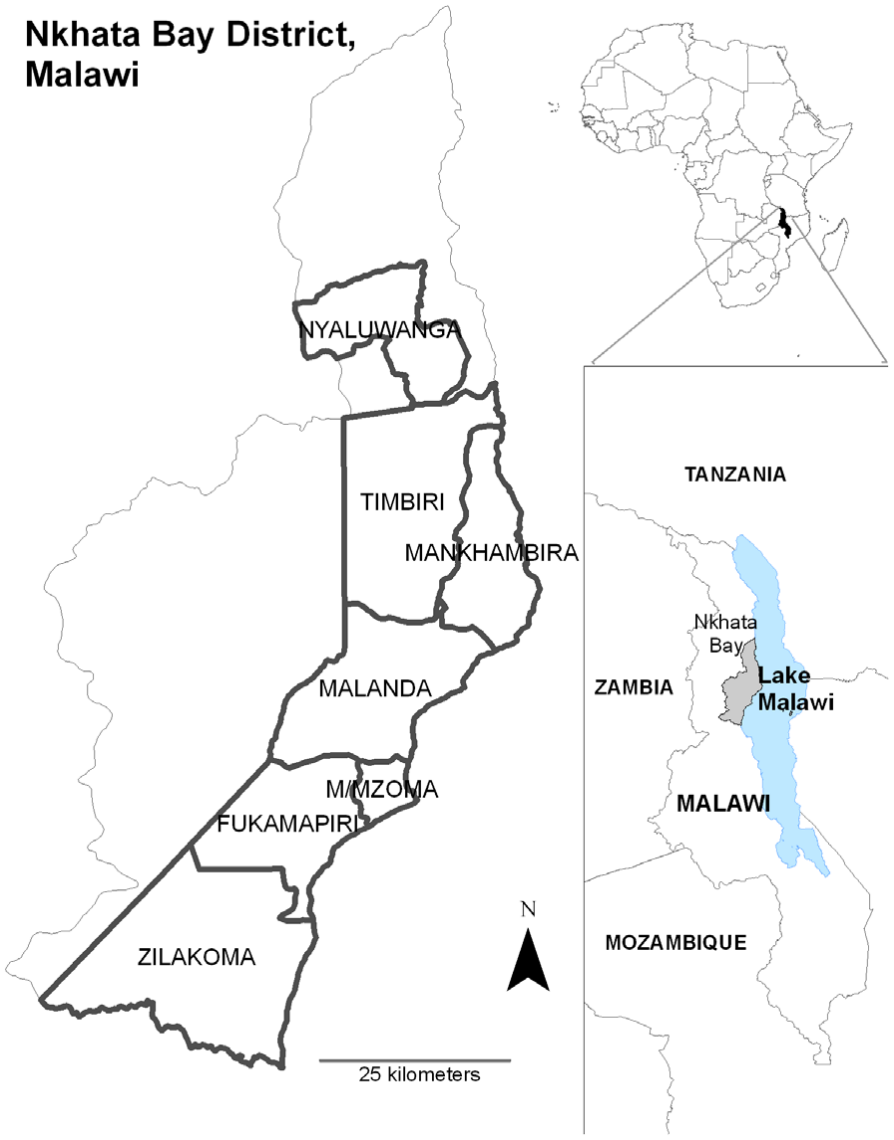
Household interviews were conducted to catalog the consequential lightning strikes in seven Traditional Authorities (TAs) of Nkhata Bay District, fringing the western shore of Lake Malawi. TA boundaries correspond to the period of 2008; Mankhambira TA shown here overlaps the urban TA of Mkumbira (not pictured), which comprises Nkhata Bay Town but was not surveyed. During sampling, Fukamalaza was combined with Malanda TA.

We identified 225 CLS resulting in 454 victims (1–8 victims/strike) between 1979 (the earliest remembered event) and 2010 in Nkhata Bay District, Northern Province, Malawi (Fig. 1). Of 450 victims with known outcome, 61% resulted in unconsciousness, 8% unconsciousness with other injuries, 3% injury alone, and 26% in death (referring to immediate outcome not to differential care or medical treatment). Data on the long-term outcome of 317 survivors revealed that 85% made a full recovery, although permanent symptoms included hearing damage (8%), headaches (3%), other lasting pain (3%), and psychological damage (2%). Strikes occurred during day and night (before midnight) but mostly in the wet season.

Preponderance of reports of CLS victims from the last three years (mean annual CLS 2007–2010 = 56.3; 1979–2007 = 10.2) likely reflects under-reporting of earlier cases due to outmigration after trauma. In rural Africa families struck by misfortune commonly attribute inexplicable events to enmity, conflict, or malfeasance, and tensions are frequently solved by moving away. Alternative explanations for recent high victim rates include memory erosion (unlikely given vivid and detailed accounts of CLS in almost every interview); a rapid increase in lightning strikes since 2007 (difficult to evaluate, but unlikely); changes in activity patterns that put people at greater exposure (again, difficult to evaluate, but unlikely given persistence of subsistence agricultural livelihoods); or a precipitous increase in number of people at risk – also unlikely given current population growth rates in Nkhata Bay District of 2.8% [4].

Relying on recent CLS and contemporary census data [4] we calculate that between October 2007 and September 2010, the annual average rate of *being injured* from a lightning strike was 327/million (range 25–1,026/million depending on TA). The annual average rate for being *killed* was 84/million (0–174/million).

## Discussion

**Figure 2:**
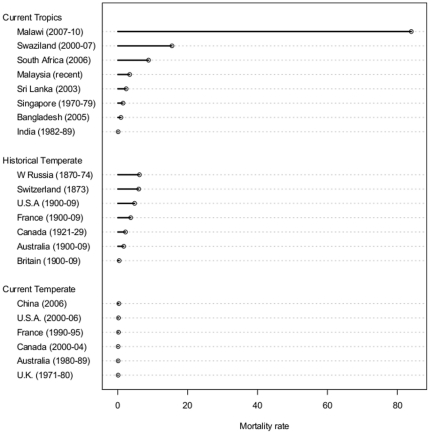
Estimates of mortality from lightning per million people per annum. Data are taken from Holle [1] and were compiled from published estimates, except for Malawi (this study) and Swaziland [5]. Selected data include historical estimates to approximate rates in 1900 (+/− 30 yrs). All mortality rates reported for current temperate countries are less than 1.0 per million people per annum.

Annual lightning-caused mortality rates are estimated as 6/million for the developing world [1] with 15.5/million being reported for Swaziland [5]. The highest mortality rate measured in our Nkhata Bay study area (Fukamapiri TA) is 11 times greater than that of Swaziland, with the mean rate recorded for Nkhata Bay District being 5.5 times greater than the Swaziland national rate. A 10∶1 injuries∶death ratio [6] gives an estimated average annual rate of 66/million of being struck in the developing world; our measured rate in Nkhata Bay was 419, greater by six times. Comparing this rate to recent and historic estimates of lightning mortality compiled by Holle [1] demonstrates the substantial relative risk in our study site (Fig. 2), as well as the trend of decreasing lightning mortality with increasing economic development seen in Europe and the USA [7]. These remarkable figures likely reflect both the proximity of Lake Malawi, Africa's third largest lake (30,000 km^2^ and 600 km long, known as the Lake of Storms [8]) and our reliance on community level reports rather than official (typically underestimated) figures [1]. Our study area on the northwest shore protrudes into the lake and appears prone to disproportionate CLS from storms over the lake.

Where the risk of extrinsic mortality is great there are diminishing returns to parental effort [9], [10], which selects for high fertility and lower investment in existing offspring [11]. For humans there is some evidence of links between the risk of extrinsic mortality and life history traits. For example, giving birth at an early age is linked to high crime rates in the USA [12], to high rates of HIV infection in South Africa [13], and to low life expectancy in the UK [14]; moreover, cross cultural analyses suggest that populations experiencing famine and/or warfare show lower levels of some components of parental care, such as sleeping proximity [15]. We might therefore expect less diligent child care, higher fertility and earlier age of first reproduction in areas experiencing high CLS than in areas with lower lightning activity, other factors being equal. Furthermore, cases of apparently random death, as occur with lightning strikes, can elicit accusations of witchcraft against community members with whom relationships are strained, such as neighbours with property conflicts or unpaid debts. Such outcomes often lead to one or more families moving out of the area, compounding the emotional loss with economic and social costs.

Reduction in risks of CLS in developed nations arose through urbanization and changing building construction [16]. If risk of lightning in northern Malawi represents as substantial a threat as our data indicate, direct intervention may be required. Possible routes entail lightning conductors on buildings, improved shelters, and greater awareness [17]. Specifically, we suggest that District Governments adjacent to the Great Lakes Region of East and Central Africa instigate programmes of increasing public awareness through workshops and displaying lightning safety instructions in schools and on beaches. In particular, the importance of staying inside should be stressed, as well as remaining under cover if caught outside, since these seemed to have effects on incidences of CLS. Certainly, specialists should be consulted with respect to the merits of placing lightning conductors on buildings such as schools and churches where people collect.

In relation to national and international development strategies, however, caution is required. It is important to stress that rates of mortality during infancy and from diseases including malaria, TB, and HIV-AIDS are far greater threats to community welfare than being struck by lightning [18]. As an anecdote, Livingstone's successors abandoned Old Bandawe because of malaria rather than lightning as the gravestones there attest. Our findings regarding lightning death and injury should not deflect resources away from the prevention of more common causes of mortality, which are being tackled by governments, development and civil society organizations throughout the developing world.

## Methods

Following local usage we define victims as individuals present at the lightning strike and rendered unconscious, injured, or dead. To determine the prevalence of fatal and non-fatal lightning strikes, we conducted a comprehensive cross-sectional survey of all consequential lightning strikes (CLS) across seven Traditional Authorities (TA), comprising 134,379 total people [4], in Nkhata Bay District (Northern Province, Malawi) over three periods between June 2009 and June 2011. The TAs were Fukamapiri, Malenga Mzoma, Mankhambira, Malanda, Nyaluwanga, Timbiri and Zilakoma (Fig. 1). Structured interviews were administered directly to victims/survivors and witnesses in order to avoid underreporting inherent in death certificates, newspaper articles, and hospital discharge data [16]. Following the initial survey period (June–August 2009) covering five TAs surrounding Nkhata Bay Town, the second survey (September–November 2010) was used to update the record of incidences in this area, and to determine the extent of missed reports in the previous survey; a further 15 cases of CLS from 1979–2009 were uncovered in three of the TAs believed to have been completed in 2009, suggesting under-reporting in the initial survey of at least 5%. The third survey period (May–June 2011) expanded the study site to include adjacent TAs to the north and south along the lake shore, Nyaluwanga and Zilakoma. Injuries and deaths per person/annum were calculated for each TA, and for the seven TAs combined, using government census data [4].

Mean annual rainfall is 1524 mm at Nkhata Bay, nearly 90% of which falls December to March [19], and elevation rises from 500m at the lakeshore to 1,700 m. To avoid underreporting [16] we worked with local Malawian male interviewers from the area who employed snowball sampling and who worked separately. Upon arriving in a TA, they met with the traditional chief and assistants to make an initial list of CLS. Subjects were then visited in their homes, introduced to the aim of the study, the nature of the questions, and the means of withdrawal. The study design allowed for incidents to be recorded without pursuit of further details from affected subjects. Consent was verbal because of low levels of literacy, and documented through the subjects' agreement to answer a questionnaire on arrival at the household. In reported cases where the affected household had been abandoned after the strike and the family had left the area, details were taken from neighbors/witnesses where possible. Before leaving each household, interviewers asked for any known further cases, adding these to their list until no new cases were uncovered. The verbal consent process was approved by the Malawian authorities. No request for a waiver of the consent process was made to the UC Davis IRB committee (as explained below).

Ethical approval was not requested from the University of California Davis, but full approval was attained from the district government office in Malawi. There are four reasons why approval from UCD was not requested – the study was requested by the Malawian authorities in Nkhata Bay, risks to subjects were judged to be minimal, data collected were entirely public, and strike victims and witnesses were identified through public knowledge annulling issues of anonymity. Research assistants were familiar with the etiquette of dealing with sensitive issues in Lakeside Tonga culture. They sought introductions to affected households from friends and neighbours and gave household members full assurance that interviews were voluntary and/or could be scheduled at another time; research assistants were also permitted to write up responses only *after* leaving the household, in cases where subjects wished to talk informally about the event. These techniques served to minimize stress, increase trust, and (we believe) enhance the reliability of the data.
